# Enhancing the immunogenicity of tumour lysate-loaded dendritic cell vaccines by conjugation to virus-like particles

**DOI:** 10.1038/bjc.2011.538

**Published:** 2011-12-01

**Authors:** S J Win, D G G McMillan, F Errington-Mais, V K Ward, S L Young, M A Baird, A A Melcher

**Affiliations:** 1 Department of Microbiology and Immunology, Otago School of Medical Sciences, University of Otago, Dunedin, New Zealand; 2 Division of Oncology, Leeds Institute of Molecular Medicine, University of Leeds, Wellcome Trust Brenner Building, Leeds LS9 7TF, UK; 3 Institute of Membrane and Systems Biology, Faculty of Biological Sciences, University of Leeds, Leeds, UK; 4 Department of Pathology, University of Otago, Dunedin, New Zealand

**Keywords:** VLP, tumour immunotherapy, lysate therapy

## Abstract

**Background:**

Tumour cell lysates are an excellent source of many defined and undefined tumour antigens and have been used clinically in immunotherapeutic regimes but with limited success.

**Methods:**

We conjugated Mel888 melanoma lysates to rabbit haemorrhagic disease virus virus-like particles (VLP), which can act as vehicles to deliver multiple tumour epitopes to dendritic cells (DC) to effectively activate antitumour responses.

**Results:**

Virus-like particles did not stimulate the phenotypic maturation of DC although, the conjugation of lysates to VLP (VLP-lysate) did overcome lysate-induced suppression of DC activation. Lysate-conjugated VLP enhanced delivery of antigenic proteins to DC, while the co-delivery of VLP-lysates with OK432 resulted in cross-priming of naïve T cells, with expansion of a MART1^+^ population of CD8^+^ T cells and generation of a specific cytotoxic response against Mel888 tumour cell targets. The responses generated with VLP-lysate and OK432 were superior to those stimulated by unconjugated lysate with OK432.

**Conclusion:**

Collectively, these results show that the combination of VLP-lysate with OK432 delivered to DC overcomes the suppressive effects of lysates, and enables priming of naïve T cells with superior ability to specifically kill their target tumour cells.

Immunotherapy of melanomas is an area of great current clinical interest, owing to the immunogenic nature of melanomas and the identification of many defined tumour antigens (Kawakami *et al*, 1994; Romero *et al*, 2002). Many therapeutic strategies have centred around the activation of cytotoxic T lymphocytes (CTLs) to produce high levels of IFN-*γ* and specific cytolytic activity. Strategies for inducing tumour-specific CTL *in vivo* have utilised adjuvant-activated dendritic cells (DC) pulsed with melanoma peptides or tumour lysates, either delivered directly to patients or used for *ex vivo* activation of autologous T cells for adoptive transfer (Dudley *et al*, 2005; Fang *et al*, 2008). Although these approaches have been used in clinical trials, they have yielded modest benefits for patients to date, suggesting that modifications to these strategies may improve the specificity and potency of antitumour immune responses (Hershey *et al*, 2002; Salcedo *et al*, 2006; Hong *et al*, 2010).

The use of DC pulsed with tumour lysates and activated with an adjuvant is thought to result in limited responses because of the induction of tolerance in the context of DC–tumour interactions (Mehrotra *et al*, 2003). This may in part be due to the inhibitory nature of lysates on the concomitant activation of DC as, for example, DC pulsed with lysates and matured with the potent bacterial adjuvant OK432 have been shown to secrete less cytokines and have lower expression of MHC-II and costimulatory molecules than unpulsed DC matured with OK432 (Hatfield *et al*, 2008).

Virus-like particles (VLP) are a candidate vaccine with the potential to enhance the immunogenicity of tumour lysate proteins, as they can act as vehicles for the delivery of heterologous antigens to DC, leading to the generation of strong cytotoxic T-cell responses (Storni *et al*, 2004; Qiao *et al*, 2006). Virus-like particles are formed from viral capsid proteins and are stable, non-replicative and highly immunogenic, making them a safe option for the treatment of cancer patients. Model tumour antigens either chemically coupled to rabbit haemorrhagic disease virus virus-like particles (RHDV VLP) or recombinantly expressed within the particles, have shown strong efficacy *in vivo* using murine models of melanoma (Peacey *et al*, 2008).

Previous studies have demonstrated that RHDV VLP conjugated to tumour lysates are cross-presented by human DC to induce strong CD8^+^ T-cell responses *in vitro*, although, murine DC have shown no evidence of activation by VLP (Win *et al*, 2011). Cross-presentation of tumour antigens is an important prerequisite for the development of activated T cells with cytotoxicity against tumour cells, although DC induce more potent cytotoxic T-cell responses when they have been optimally matured (Storni *et al*, 2002; Keller *et al*, 2010).

Here, we demonstrate that VLP conjugated to tumour cell lysates are able to induce specific immune responses towards tumour cells, while negating the inhibitory effects of lysates delivered alone. This is achieved through enhanced delivery of antigen and cross-presentation capabilities of DC, and is further dependent on the addition of an adjuvant to ensure effective DC maturation and priming of naïve T-cell responses.

## Materials And Methods

### Generation and coupling of Mel888 lysates to VLP

Conjugation of Mel888 tumour cell lysates to RHDV VLP was carried out as previously described (Peacey *et al*, 2007; Win *et al*, 2011). Mel888 tumour cells (Cancer Research UK) were grown in DMEM with 10% FCS and 1% L-glutamine and lysates were made by three repeat cycles of freeze–thaw lysis in the presence of complete mini EDTA-free protease inhibitor tablets (Roche Diagnostics Ltd, Mt Wellington, New Zealand). Cells were routinely tested for *Mycoplasma* and found to be free of infection.

Lysates were mixed with a 10-fold molar excess of the heterobifunctional linker sulfo-succinimidyl 4-(*N*-maleimidomethyl)cyclohexane-1-carboxylate (sulfo-SMCC) (Pierce, Rockford, IL, USA) in phosphate buffer pH 7.3 followed by dialysis to remove unbound chemical linker. Virus-like particle were reacted with a 10-fold molar excess of *N*-succinimidyl *S*-acetylthioacetate (SATA) (Pierce) followed by treatment with 100 *μ*g hydroxylamine.HCl (Sigma, Dorset, UK) per ml of protein. Thiol-activated VLP were passed through a HiTrap desalt column (GE Healthcare, Bucks, UK) to remove unbound SATA. Equal concentrations of thiol-activated VLP and malemide-activated lysates were mixed together for 1 h. Coupling was confirmed using sodium dodecyl sulphate-polyacrylamide gel (SDS–PAGE) containing 10% or 15% acrylamide and western blotting using polyclonal rabbit anti-VLP and monoclonal human anti-MART-1 antibodies (clone M2-7C10; Serotec, Oxon, UK).

### Preparation of monocyte-derived DCs

The white blood cell fraction from apheresis blood products (National Blood Service, Leeds, UK) was separated using Lymphoprep (Axis-Shield, Dundee, Scotland, UK) gradients and CD14^+^ monocytes were selected with a MACS column using anti-CD14 magnetic beads (Miltenyi Biotec, Surrey, UK). The CD14^+^ cells were cultured for 5 days in RPMI (Sigma) containing 10% FCS with 800 U ml^–1^ GM-CSF and 500 U ml^–1^ IL-4 (R&D Systems, Abingdon, UK) at 37 °C+5% CO_2_ (Errington *et al*, 2008).

### DC activation

Dendritic cells were exposed to 50 *μ*g ml^–1^ VLP-Mel888 lysates, Mel888 lysates or left un-stimulated for 4 h before the addition of 10 *μ*g ml^–1^ OK432 (Chugai Pharmaceutical Co., Ltd, Tokyo, Japan) for 24 h at 37 °C+5% CO_2_. Cell staining was performed using anti-CD11c-APC and PE-conjugated monoclonal antibodies directed against epitopes of CD40, CD80, CD86 and HLA-DR. Dendritic cells were incubated with the respective antibodies or IgG isotype control antibodies and fluorescence was measured using a FACSCalibur (BD Biosciences, Oxford, UK). Supernatants were collected and assayed for the presence of IL-12p70, CCL5, IL-6 and TNF-*α* by ELISA using matched antibodies (BD Biosciences).

### Subcellular fractionation

Dendritic cells (2 × 10^6^ ml^–1^) were incubated with VLP, Mel888 lysate or VLP-Mel888 lysate (50 *μ*g ml^–1^) for either 1 or 3 h. After three washes in ice-cold PBS (pH 6.8), cell pellets were re-suspended in 1 ml homogenisation buffer (HB; PBS, 0.25 M sucrose, 10 mM Tris, 1 mM EDTA with protease inhibitors and DNase (Complete – Roche Diagnostics, Sussex, UK), pH 6.8) and gently homogenised with a cell-cracker (HGM laboratory equipment). Postnuclear supernatant was prepared by centrifugation of crude lysis at 1000 × **g** for 10 min at 4 °C. The supernatant was moved to a new microfuge tube. Light membranes (recycling and early endosomes) were separated from heavy membranes (late endosomes and lysosomes) by centrifugation at 8000 × **g** for 20 min at 4 °C. The post-8000 **g** supernatant was adjusted to 40% (w/v) sucrose using a stock of 80% (w/v) sucrose in HB to a final volume of 4 ml. This was overlayed with a 4 ml volume of 35% (w/v) sucrose in HB, followed by a 4 ml volume of 5% (w/v) sucrose in HB. Recycling endosomes were separated from early endosomes by ultracentrifugation at 180 000 × **g** for 16 h at 4 °C. The top 3 ml was discarded. The next 5 ml (recycling endosomes) was washed with PBS (pH 6.8) and pelleted by ultracentrifugation at 144 651 × **g** for 2 h at 4 °C. The last 4 ml from the separation (early endosomes) was washed with PBS (pH 6.8) and pelleted by ultracentrifugation at 144 651 × **g** for 2 h at 4 °C. The post-8000 **g** pellet was re-suspended in 4 ml HB containing 10% Percoll and loaded on top of 6 ml of HB containing 45% Percoll. Late endosomes and lysosomes were separated by ultracentrifugation at 50 000 × **g** for 1 h at 4 °C. The top 4 ml (late endosomes) was washed with PBS (pH 6.8) and pelleted by ultracentrifugation at 144 651 × **g** for 2 h at 4 °C. The bottom 4 ml (lysosomes–late endosome fusions) was washed with PBS (pH 6.8) and pelleted by ultracentrifugation at 144 651 × **g** for 2 h at 4 °C.

### SDS–PAGE and immunoblotting

Cellular fractions were routinely analysed on 12.5% SDS–PAGE in the presence of 0.1% SDS using the buffer system of Laemmli (1970). Polypeptide bands were visualised using Simply Blue Safe Stain (Invitrogen, Paisley, UK). During immunoblotting, membrane vesicles were subjected to 12.5% SDS–PAGE followed by electroblotting onto a polyvinylidene difluoride membrane including 0.02% SDS in the running buffer. Cell fractions were probed using rabbit-derived serum against RHDV-VLP and antibodies against MART1 (Serotec), LAMP-1 (clone 25, BD Biosciences), EEA1 (clone 14, BD Biosciences), Rab11 (clone 47, BD Biosciences) and Rab7 (clone 117, Abcam, Cambridge, UK). Horseradish peroxidase-conjugated goat-derived anti-mouse secondary antibody was used for detection (Invitrogen). The antibody-specific bands were visualised using the SuperSignal West Pico chemiluminescence system (Pierce).

### Protein assay

Protein concentrations were determined using a bicinchoninic acid protein assay kit (Sigma) with bovine serum albumin as the standard.

### Naïve T-cell priming

HLA-A2^+^ DC (1 × 10^6^ cells ml^–1^) were harvested and pulsed with 50 *μ*g ml^–1^ VLP, Mel888 lysate or VLP-Mel888 lysate for 4 h before the addition of 10 *μ*g ml^–1^ OK432 overnight. The DC were washed and cultured with PBMC at a ratio of 15–40 : 1 (PBMC:DC) in CTL media (RPMI with 1% non-essential amino acids, 1% L-glutamine, 1% sodium pyruvate, 1% HEPES, 20 *μ*M 2-mercaptoethanol and 7.5% human serum (Sigma)) containing 10 *μ*g ml^–1^ IL-7 (R&D Systems) for 7 days at 37 °C+5% CO_2_. After 4 days, 30 U ml^–1^ of IL-2 (R&D Systems) was added to the CTL cultures. After 7 days, fresh DC pulsed with VLP, Mel888 lysate or VLP-Mel888 lysate were added and cultured for a further 7 days. The CTL were then assessed for their specificity and cytotoxic activity against Mel888 target cells with ovarian tumour cells, SKOV-3, used as irrelevant target cells.

### MART1 pentamer staining

CTL (1 × 10^6^ cells) were labelled with APC-conjugated ELAGIGILTV pentamer (F082-4A, ProImmune, Oxford, UK), FITC-conjugated anti-CD8 and PE-conjugated anti-CD19 antibodies or respective IgG isotype controls (BD Biosciences). The cells were analysed by flow cytometry.

### Cytotoxicity assay

T-cell cytotoxicity was measured by ^51^Cr release where target cells (5000 cells per well) were labelled with 100 *μ*Ci ^51^Cr (Perkin Elmer, Cambridge, UK) for 1 h then washed three times in RPMI containing 10% FCS and 1% L-glutamine. Labelled cells were then incubated with cold Daudi cells (5000 cells per well, to quench nonspecific killing) and CTL at different effector:target ratios. After 4 h, the cells were pelleted and 50 *μ*l of supernatant was transferred to scintillation plates (Perkin Elmer) and left to dry overnight. Levels of ^51^Cr were measured in the supernatants using a Wallac Jet 1459 Microbeta Scintillation counter and Microbeta Windows software (Perkin Elmer).

Percent specific lysis was calculated by the following formula:



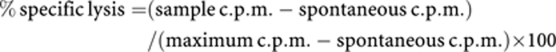



### CD107 and IFN-*γ* release

Cytotoxic T lymphocytes (2.5 × 10^5^ cells) were cultured with target cells at a 1 : 1 ratio for 1 h at 37 °C before the addition of FITC-conjugated anti-CD107a and CD107b antibodies (BD Biosciences), and 10 *μ*g ml^–1^ brefeldin A (Sigma) or 10 *μ*g ml^–1^ brefeldin A only. The cells were incubated for a further 4 h before co-staining with PerCP-conjugated anti-CD8 antibodies (BD Bioscences) for 30 min. The CD107a/b-labelled cells were fixed in 1% paraformaldehyde (Sigma) and analysed by flow cytometry while the additional set were stained for intracellular IFN-*γ* (BD Bioscences). These cells were fixed in 1% paraformaldehyde before permeabilising in 0.3% saponin (Sigma) for 30 min and stained with FITC-conjugated anti-IFN-*γ*. The cells were washed and analysed immediately by flow cytometry.

### Statistical analyses

Student's two-tailed paired *t*-tests were performed where statistics are shown using GraphPad Prism version 5.0b (La Jolla, CA, USA). Significance was assigned where *P*-values were 0.05 or less.

## Results

### RHDV VLP require the addition of an adjuvant to mature DC, although conjugation of tumour cell lysates to VLP overcomes the inhibitory effects of lysates on DC maturation

Activation of cytotoxic T-cell responses requires DC to deliver antigen-specific signals to T cells by way of MHC-I:peptide ligation of the TCR, while also providing costimulatory molecule engagement and secretion of appropriate cytokines. Only upon maturation, or activation, do DC provide the necessary levels of costimulation and cytokines to induce activation and expansion of their cognate T cells (Mescher *et al*, 2006).

Previous studies demonstrated that RHDV VLP do not result in the phenotypic activation of DC *in vitro,* so we also included an adjuvant to enhance the antigen presentation capacity of VLP-lysate-loaded DC. OK432 was chosen as a suitable adjuvant, as it has been shown to activate human monocyte-derived DC and is licensed for clinical use (Hovden *et al*, 2011). OK432 is a preparation of a penicillin-killed, low virulence strain of streptococcus pyogenes (group A) and has been used as an immunotherapeutic agent in a number of malignancies without significant clinical side effects. The antitumour effects of OK432 are thought to be mediated by the activation of a variety of effector cells, presumably including DC (Kuroki *et al*, 2003).

**Figure 1:**
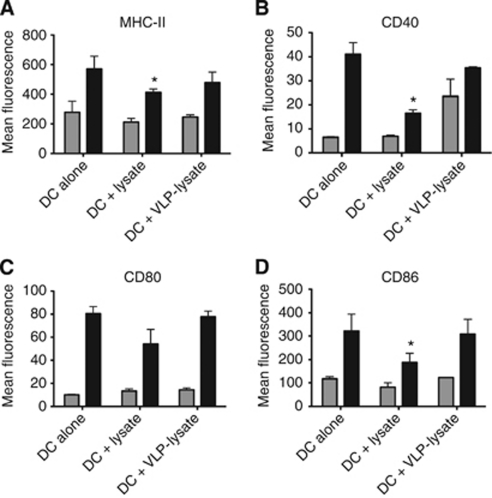
Conjugation of tumour lysates to VLP negates their inhibitory effects DC phenotypic maturation. Bar graphs represent (**A**) MHC-II, (**B**) CD40, (**C**) CD80 and (**D**) CD86 expressed on DC alone or pulsed with Mel888 Lysates (Lysate) or VLP-Mel888 Lysates (VLP-Lysate) 4 h prior to the addition of no adjuvant (grey bars) or OK432 (black bars) for 24 h. Data represents mean±SD for 6 individual donors. *Denotes statistical significance (*P*<0.05) between lysate-pulsed and unpulsed DC.

**Figure 2:**
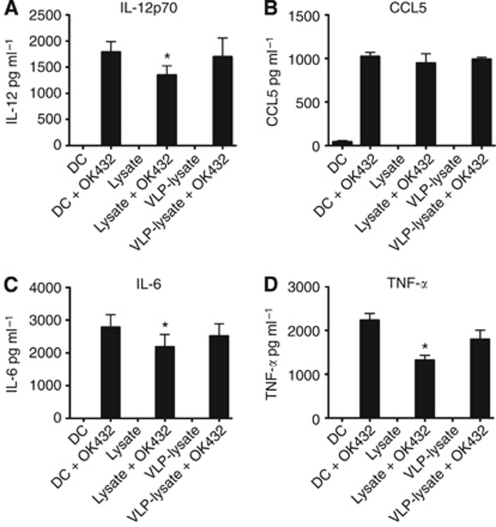
Conjugation of tumour lysates to VLP negates their inhibitory effects on DC cytokine/chemokine production. Bar graphs represent (**A**) IL-12p70, (**B**) CCL5, (**C**) IL-6 and (**D**) TNF-*α* produced by DC after pulsing with lysate, VLP-lysate or left unpulsed, before the addition of OK432 or no adjuvant. Data represent mean±s.d. for six experiments. ^*^Denotes statistical significance (*P*<0.05) between lysate-pulsed and unpulsed DC.

Human DC were pulsed with VLP conjugated to Mel888 lysates (VLP-lysate) or lysates, either alone or with OK432 before staining for surface markers of activation (Figure 1) and determining the cytokine secretion profiles of these cells (Figure 2).

Dendritic cells pulsed with VLP-lysate or lysates without adjuvant did not activate DC to increase expression of MHC-II, CD40, CD80 or CD86, nor did they induce secretion of the proinflammatory cytokines IL-12, IL-6 and TNF-*α* or the chemokine CCL5. Conversely, the addition of OK432 induced clear increases in MHC-II and costimulatory molecules on the surface of the DC, while also inducing high levels of proinflammatory cytokine and CCL5 secretion.

Interestingly, lysates delivered with OK432 resulted in decreased MHC-II and costimulatory molecule expression and cytokines compared with DC pulsed with OK432 alone. We have previously found this inhibitory effect of lysates on adjuvant-induced activation of DC in both murine and human systems (Hatfield *et al*, 2008; unpublished data). However, this inhibition was not seen when VLP were conjugated to the lysates, suggesting that the inhibitory effects of lysates on adjuvant-induced activation of DC is reversed by the presence of VLP.

### Conjugation of tumour cell lysates to VLP enables more efficient delivery of tumour antigens to DC compartments for processing

One of the requirements of successful tumour immunotherapy is ensuring efficient delivery of therapeutic antigens to DC in a manner resulting in epitope presentation conducive to the development of cytotoxic T-cell responses. Previous studies have demonstrated that immunogenic peptides or tumour cell lysates conjugated to VLP are cross-presented to CD8^+^ T cells by DC more effectively than peptide or lysates delivered alone; however, the reasons underpinning this effect are not well defined (Win *et al*, 2011). To further understand the efficiency of antigen delivery and the intracellular trafficking of these antigens, DC pulsed with lysates, VLP or lysates conjugated to VLP were fractionated into their constituent subcellular compartments and probed for the presence of VLP or lysate.

**Figure 3:**
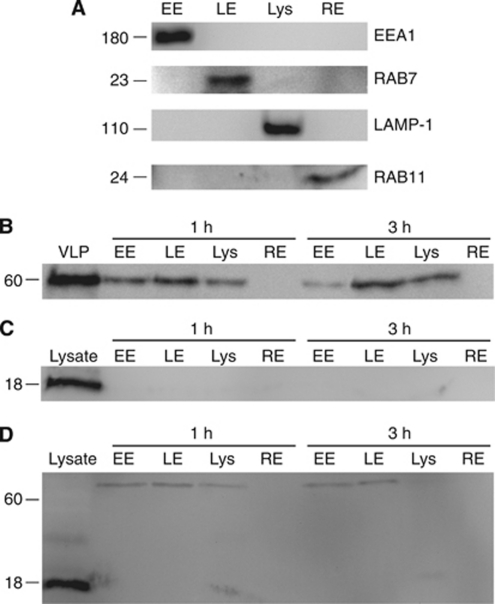
Conjugation of lysates to VLP enhances delivery of tumour antigens to DC antigen processing compartments. (**A**) Subcellular fractions containing early endosomes (EE), late endosomes (LE), lysosomes (Lys) or recycling endosomes (RE) were prepared from DC. The following probes were used as markers of specific subcellular compartments; EEA1 (EE), Rab7 (LE), LAMP1 (Lys) and Rab11 (RE). (**B**) Dendritic cells were incubated with VLP for 1 or 3 h. After washing, subcellular fractions were prepared and the presence of VLP was analysed by western blot using anti-VP60 antibodies. (**C**) Dendritic cells were incubated with lysate for 1 or 3 h. After washing, subcellular fractions were prepared and the presence of MART1 was analysed by western blot. (**D**) Dendritic cells were incubated with VLP-lysate for 1 or 3 h. After washing, subcellular fractions were prepared and the presence of MART1 was analysed by western blot. Results are representative of four independent experiments.

To confirm the subcellular compartments, an initial DC fractionation was carried out separating early endosomes (EE), late endosomes (LE), lysosomes (Lys) and recycling endosomes (RE) into discrete compartments (Figure 3A). Western blot analysis identified a single band for each of EEA1, Rab7, LAMP1 or Rab11, defining the EE, LE, Lys and RE compartments respectively.

Dendritic cells were then pulsed with VLP, VLP-lysate or lysate alone for 1 or 3 h, and washed to remove excess antigen. The DC were subjected to fractionation into their constituent subcellular compartments and probed by western blot for the presence of the VLP VP60 protein or the Mel888 lysate protein MART1 (Figures 3B–D). The resultant blots show that VLP were delivered effectively to DC, as entire VP60 protein was present in EE, LE and Lys, but not RE, after both 1 and 3 h. Interestingly, no MART1 was detected in any of the endosomal fractions at either time point when lysates delivered alone were the source of antigen; however, MART1 from VLP-conjugated lysates was detected in large molecular weight complexes at both 1 and 3 h after delivery to DC. MART1 was present in both EE and LE, and to a lesser degree Lys, at both time points. Additionally, at 1 h a small band of 20–30 kDa was seen in the lysosomal fraction, indicating degradation of the higher molecular weight MART1-containing complexes into single MART1 molecules. It is likely that reduced amounts of protein being detected in Lys, and none in RE, is due to degraded forms of the VP60 or MART1 proteins being undetectable by their antibodies. These results indicate that conjugation of lysates to VLP enables more efficient delivery and retention of the antigenic proteins in antigen processing compartments by DC, than lysates delivered alone.

### Delivery of VLP conjugated to tumour cell lysates together with an adjuvant enables cross-priming of naïve CD8^+^ T-cell responses

Activation and expansion of T cells expressing TCRs specific for melanoma antigens from circulating naïve T cells is essential for achieving adaptive cytolytic activity against tumour cells, while ensuring that tumour cells are the exclusive targets of any stimulated CTL. Activating CTL against one melanoma epitope, using single peptide therapies, can achieve significant death of target tumour cells, but can also lead to tumour escape whereby tumour cells that do not express that specific antigen continue to grow (Sanchez-Perez *et al*, 2005). Thus, it is desirable to incorporate many tumour antigens into a DC-based vaccine, so CTL that recognise diverse tumour epitopes can be activated and expanded to prevent tumour escape. To achieve this, VLP conjugated to Mel888 tumour cell lysates or lysates alone were pulsed onto DC with the adjuvant OK432, or with no maturation stimulus and used to prime naïve T-cell responses against Mel888 tumour cells using established methodology (Errington *et al*, 2008).

**Figure 4:**
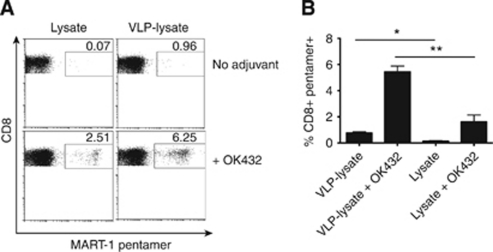
Expansion of MART1-specific T cells following successive rounds of priming with VLP-lysate or lysate in the presence or absence of adjuvant. (**A**) Plots demonstrate CD8^+^ T cells expressing a T-cell receptor specific for MART1 from one representative experiment. (**B**) Bars represent mean±s.d. for six experiments. ^*^ and ^**^ denotes statistical significance (*P*<0.05 and <0.01) between VLP-lysate or lysate-primed CTL without or with OK432.

**Figure 5:**
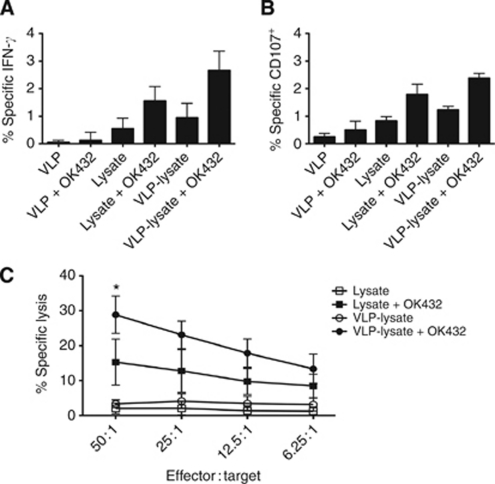
Activity of T cells following priming with VLP-lysate or lysates in the presence or absence of adjuvant. (**A**) Intracellular IFN-*γ* staining within CD8^+^ T cells. (**B**) Surface CD107 expression on CD8^+^ T cells. Bars represent specific IFN-*γ* production or CD107 degranulation on co-culture with Mel888 targets with values against irrelevant SKOV-3 targets subtracted. (**C**) Cytotoxicity of primed T cells against Mel888 targets labelled with ^51^Cr. Nonspecific lytic activity against irrelevant ^51^Cr-labelled SKOV-3 targets has been subtracted. Data represent mean±s.d. for six experiments, ^*^Denotes statistical significance (*P*<0.05) between VLP-lysate+OK432 and lysate+OK432.

To determine the expansion of CTL toward a defined melanoma antigen, the primed cells were stained with a MART-1 pentamer. This demonstrated that antigen-specific T cells were successfully expanded when VLP-lysate or lysates were pulsed onto DC with OK432 (Figure 4). The use of VLP-lysate with OK432 led to greater expansion of MART1-specific T cells than loading DC with unconjugated lysates and OK432. Moreover, VLP-lysate/OK432 primed a population of CTL able to specifically degranulate against, produce IFN-*γ* on recognition of, and directly lyse Mel888 tumour cells to a greater degree than unconjugated lysates/OK432 (Figure 5). Interestingly, the amount of unconjugated lysate pulsed on to DC (50 *μ*g ml^–1^) was the same amount as total VLP-lysate added where VP60 protein constitutes at the very least half of the total protein delivered. Although it is not well defined exactly which proteins are bound to the VLP, it is likely that the efficiency of this coupling and size constraints limit conjugation of lysate proteins, such that lysate delivered on VLP equates quantitatively to less tumour antigenic protein than pulsing DC with unconjugated lysate by at least half. This suggests that VLP-lysates are particularly efficient at delivering antigens to DC for cross-presentation to T cells.

## Discussion

Virus-like particles carrying tumour antigens are able to improve the survival and delay the growth of tumours in mice with concomitant *in vivo* cytotoxicity (Peacey *et al*, 2008). However, it is crucial to translate these findings into human systems to further understand how human immune cells may interact with VLP to generate cytotoxic responses effective in melanoma patients.

Dendritic cells pulsed with VLP conjugated to tumour lysates did not result in phenotypic activation or proinflammatory cytokine secretion above the levels seen with unpulsed DC. This may be due to the lack of nucleic acid within the VLP acting as a pathogen-associated molecular pattern used by cells to recognise viruses through ligation of pattern recognition receptors. However, the addition of an adjuvant, streptococcal-derived OK432, induced maturation of DC with secretion of the proinflammatory cytokines that favour the development of a Th1, cytotoxic immune response. Consistent with current literature in a murine model (Hatfield *et al*, 2008), the use of lysates derived from tumour cells by way of freeze and thaw cycles was shown to be inhibitory to DC, an effect that is only partially restored with the addition of a maturation stimulus (note in Figures 1 and 2 the reduced activation of DC by OK432 in the presence, compared with the absence, of lysate). However, the mechanism(s) responsible for this lysate inhibition have yet to be fully elucidated. It has been disputed whether a particular mode of cell death will lead to immune cell activation or suppression, with many reports suggesting that necrotic cell death, similar to that seen when preparing lysates by freeze thaw, is more stimulatory than apoptosis (Strome *et al*, 2002; von Euw *et al*, 2007; Herzog *et al*, 2011). One factor missing from the ‘necrotic’ cell death induced by freeze–thaw lysis, however, is the induction of stress related molecules such as heat shock proteins, which can act as danger signals for immune activation (Flohe *et al*, 2003; Bendz *et al*, 2007; Aguilera *et al*, 2011). Therefore, the inhibitory nature of lysates may be an inevitable consequence of their method of preparation, although lysates remain highly practical as a source of multiple tumour-associated antigens for clinical application.

Interestingly, the delivery of tumour cell lysates conjugated to VLP, in contrast to unconjugated lysate, did not reduce the ability of DC to activate in the presence of OK432. There are two possible explanations for this observation. First, tumours contain many immunosuppressive molecules, which may not be preferentially coupled to VLP and second, VLP may be altering the intracellular fate of the lysate proteins coupled to their surface to facilitate their immunostimulatory access to the antigen processing pathways within DC.

To confirm that conjugation of lysates to VLP enables more effective presentation, DC were fractionated into their constituent endosomal compartments and analysed for the presence of VLP or lysate-derived proteins (MART1). The resultant blots show lysates are delivered more effectively to DC, and are retained in the endosomal system for longer, when conjugated to VLP. Consistent with our previous studies defining the mechanisms of cross-presentation of VLP by DC, the VLP and VLP-conjugated antigen were found in early and late endosomes and lysosomes, indicating that VLP are degraded into antigenic peptides within lysosomal compartments. In contrast, when lysates were delivered alone, no MART1 was detected in any endosomal fractions, despite delivering more than double the amount of lysate protein than would be conjugated to VLP. This suggests that unconjugated lysate proteins are either not taken up to a detectable level, or that these proteins are rapidly degraded by the DC, both situations resulting in reduced cross-presentation and cytotoxic responses. Recycling endosomes were not shown to contain any VLP or lysate-derived MART1, although this is not surprising, as the degradation of VP60 or MART1 into peptides would prevent the antibodies directed against the entire protein from recognising them.

Developing effective cytotoxic T-cell responses against tumour cells is one key step towards destroying them. Ensuring naïve T cells are primed for activity specifically against their tumour cell target is essential to prevent autoimmunity, while priming a pool of T cells recognising a variety of tumour antigens is important for achieving maximal tumour killing and preventing escape variants. To this effect, DC pulsed with VLP conjugated to melanoma cell lysates and matured with OK432 were able to cross-prime naïve T cells, with associated increases in the numbers of MART1-specific, IFN-*γ* producing, degranulating CD8^+^ T cells. Most importantly, these expanded CTL were cytotoxic against the tumour cells from which the lysates were originally derived. In all instances, the delivery of lysates conjugated to VLP to DC resulted in more pronounced cytotoxic T-cell responses than delivery of lysates alone, despite delivering significantly less tumour antigen on conjugation to VLP.

We propose that this superior T-cell cross-priming ability conferred by VLP is likely due to more efficient uptake than cell lysates alone, leading to a greater density of tumour associated peptides being displayed on the DC surface. Virus-like particles conjugated to antigens require endosomal processing by DC before peptide display on MHC-I, whereas peptides do not. Faure *et al* (2009) demonstrated that sustained antigen cross-presentation was observed where antigens required processing before presentation, despite less antigen being presented directly after the initial pulse (Faure *et al*, 2009). Short peptides were loaded directly onto MHC-I molecules favouring rapid presentation, but this response did not last >24 h. The long peptides that required processing were still being presented 3 days after initial pulsing, despite significant washing steps to remove any free peptides, a phenomenon potentially due to antigen storage compartments inside DC. This also may be true for VLP and their derivative peptides, as they are degraded in lysosomes before loading onto MHC-I that have recycled from the cell surface. Presumably, this exchange of peptide occurs in a post-lysosomal compartment that may serve as a reservoir for antigen cross-presentation over a sustained period of time. In the case of the lysates, we have shown that the addition of these to VLP prevents their inhibitory effects on DC activation. This together with increased delivery of lysate antigens to compartments where efficient cross-presentation can take place, may explain why T cells, which have interacted with VLP-lysate pulsed DC and matured with an adjuvant, are more effectively primed for cytotoxicity.

Tumour cell lysates have been used clinically with encouraging, though limited success (Jocham *et al*, 2004). The results presented here suggest that conjugation of tumour cell lysate proteins to VLP provides an excellent scaffold for the delivery of tumour-derived antigens to DC. The simple conjugation process is flexible enough to enable the addition of any tumour lysate to VLP quickly and efficiently, both key factors in transferring this antigen delivery platform into a potential clinical setting. The addition of an adjuvant to the DC is required for optimal priming of specific cytotoxic T-cell responses, although there remains scope for incorporating both antigen and adjuvant into the same VLP for more effective therapy and ease of use.
